# Editorial: Should Robots Have Standing? The Moral and Legal Status of Social Robots

**DOI:** 10.3389/frobt.2022.946529

**Published:** 2022-06-16

**Authors:** David J. Gunkel, Anne Gerdes, Mark Coeckelbergh

**Affiliations:** ^1^ Department of Communication, Northern Illinois University, DeKalb, IL, United States; ^2^ Department of Design and Communication, University of Southern Denmark, Odense, Denmark; ^3^ Department of Philosophy, University of Vienna, Vienna, Austria

**Keywords:** social robot, artificial intelligence, ethics, law, philosophy of technology, AI ethics, AI and society, technology policy

In a proposal issued by the European Parliament (Delvaux, 2016) it was suggested that robots might need to be considered “electronic persons” for the purposes of social and legal integration. The very idea sparked controversy, and it has been met with both enthusiasm and resistance. Underlying this disagreement, however, is an important moral/legal question: When (if ever) would it be necessary for robots, AI, or other socially interactive, autonomous systems to be provided with some level of moral and/or legal standing?

This question is important and timely because it asks about the way that robots will be incorporated into existing social organizations and systems. Typically technological objects, no matter how simple or sophisticated, are considered to be tools or instruments of human decision making and action. This instrumentalist definition (Heidegger, 1977; Feenberg, 1991; Johnson, 2006) not only has the weight of tradition behind it, but it has so far proved to be a useful method for responding to and making sense of innovation in artificial intelligence and robotics. Social robots, however, appear to confront this standard operating procedure with new and unanticipated opportunities and challenges. Following the predictions developed in the computer as social actor studies and the media equation (Reeves and Nass, 1996), users respond to these technological objects as if they were another socially situated entity. Social robots, therefore, appear to be more than just tools, occupying positions where we respond to them as another socially significant Other.

This Research Topic of *Frontiers in Robotics* seeks to make sense of the social significance and consequences of technologies that have been deliberately designed and deployed for social presence and interaction. The question that frames the issue is “Should robots have standing?” This question is derived from an agenda-setting publication in environmental law and ethics written by Christopher Stone, *Should Trees Have Standing? Toward Legal Rights for Natural Objects* (1974). In extending this mode of inquiry to social robots, contributions to this Research Topic of the journal will 1) debate whether and to what extent robots can or should have moral status and/or legal standing, 2) evaluate the benefits and the costs of recognizing social status, when it involves technological objects and artifacts, and 3) respond to and provide guidance for developing an intelligent and informed plan for the responsible integration of social robots.

In order to address these matters, we have assembled a team of fifteen researchers from across the globe and from different disciplines, who bring to this conversation a wide range of viewpoints and methods of investigation. These contributions can be grouped and organized under the following four subject areas:

## Standing and Legal Personality

Five of the essays seek to take-up and directly address the question that serves as the title to this special issue: Should robots have standing? In “Speculating About Robot Moral Standing: On the Constitution of Social Robots as Objects of Governance” Jesse De Pagter argues that the question of robot standing—even if it currently is a future-oriented concern and speculative idea—is an important point of discussion and debate in the critical study of technology. His essay therefore situates social robot in the context of anticipatory technology governance and explains how a concept like robot standing informs and can be of crucial importance to the success of this endeavor.

“Robot as Legal Person: Electronic Personhood in Robotics and Artificial Intelligence,” Brazilian jurist Avila Negri performs a cost/benefit analysis of legal proposals like that introduced by the European Parliament. In his reading of the existing documents, Avila Negri finds evidence of a legal pragmatism that seeks guidance from the precedent of corporate law but unfortunately does so without taking into account potential problems regarding the embodiment of companies and the specific function of the term “legal person” in the grammar of law.

In “Robots and AI as Legal Subjects? Disentangling the Ontological and Functional Perspective,” Bertolini and Episcopo seek to frame and formulated a more constructive method for deciding the feasibility of granting legal standing to robotic systems. Toward this end, they argue that *standing* should be strictly understood as a legal affordance such that the attribution of subjectivity to an artifact needs to be kept entirely within the domain of law, and grounded on a functional, bottom-up analysis of specific applications. Such an approach, they argue, usefully limits decisions about moral and legal status to practical concerns and legal exigencies instead of getting mired in the philosophical problems of attributing animacy or agency to artifacts.

These two efforts try to negotiate the line that distinguishes what is a thing from who is a person. Other contributions seek to challenge this mutually exclusive dichotomy by developing alternatives. In “The Virtuous Servant Owner—A Paradigm Whose Time has Come (Again),” Navon introduces a third category of entity, a kind of in between status that is already available to us in the ancient laws of slavery. Unlike other proposals that draw on Roman law, Navon formulates his alternative by turning to the writings of the Jewish philosopher Maimonides, and he focuses attention not on the legal status of the robot-slave but on the moral and legal opportunities imposed on its human master.

In “Gradient Legal Personhood for AI Systems—Painting Continental Legal Shapes Made to Fit Analytical Molds” Mocanu proposes another solution to the person/thing dichotomy that does not—at least not in name—reuse ancient laws of slavery. Instead of trying to cram robots and AI into one or the other of the mutually exclusive categories of person or thing, Mocanu proposes a gradient theory of personhood, which employs a more fine-grained spectrum of legal statuses that does not require one to make simple and limited either/or distinctions between legal subjects and objectivized things.

## Public Opinion and Perception

Deciding these matters is not something that is or even should be limited to legal scholars and moral philosophers. These are real questions that are beginning to resonate for users and non-experts. The contribution from the Dutch research team of Graaf et al. explores a seemingly simple and direct question: “Who Wants to Grant Robots Rights?” In response to this question, they survey the opinions of non-expert users concerning a set of specific rights claims that have been derived from existing international human rights documents. In the course of their survey, they find that attitudes toward granting rights to robots largely depend on the cognitive and affective capacities people *believe* robots possess or will possess in the future.

In “Protecting Sentient Artificial Intelligence: A Survey of Lay Intuitions on Standing, Personhood, and General Legal Protection,” Martínez and Winter investigate a similar question: To what extent, if any, should the law protect sentient artificial intelligence? Their study, which was conducted with adults in the United States, found that only one third of survey participants are likely to endorse granting personhood and standing to sentient AI (assuming its existence), meaning that the majority of the human subjects they surveyed are not—at least not at this point in time—in favor of granting legal protections to intelligent artifacts. These finding are consistent with an earlier study that the authors conducted in 2021 with legal professionals.

## Suffering and Moral/Legal Status

Animal rights philosophy and many animal welfare laws derive from an important conceptual innovation attributed to the English political philosopher Jeremy Bentham. For Bentham what mattered and made the difference for moral and legal standing was not the usual set of human-grade capacities, like self-consciousness, rationality, or language use. It was simply a matter of sentience: “The question is not, ‘Can they reason?’ nor, ‘Can they talk?’ but ‘Can they suffer?’” (Bentham 2005, 283). For this reason, the standard benchmark for deciding questions of moral and legal standing—a way of dividing who is a person from what remains a mere thing—is an entity’s ability to suffer or to experience pain and pleasure. And several essays leverage this method in constructing their response to the question “should robots have standing?”

In the essay “From Warranty Voids to Uprising Advocacy: Human Action and the Perceived Moral Patiency of Social Robots,” Banks employs a social scientific study to investigate human users’ perceptions of the moral status of social robots. And she finds significant evidence that people can imagine clear dynamics by which robots may be said to benefit and suffer at the hands of humans.

In “Whether to Save a Robot or a Human: On the Ethical and Legal Limits of Protections for Robots,” legal scholar Mamak investigates how this human-all-too-human proclivity for concern with robot well-being and suffering might run afoul of the law, which typically prioritizes the welfare of human subjects and even stipulates the active protection of humans over other kind of things. In effect, Mamak critically evaluates the legal contexts and consequences of the social phenomena that has been reported in empirical studies like that conducted by Banks.

And with the third essay in this subject area, “The Conflict Between People’s Urge to Punish AI and Legal Systems,” Lima et al. explore the feasibility of extending legal personhood to AI and robots by surveying human beings’ perceptions of liability and punishment. Data from their inventory identifies a conflict between the desire to punish automated agents for wrongful action and the perceived impracticability of doing so when the agent is a robot or AI lacking conscious experience.

## Relational Ethics

In both moral philosophy and law, what something is largely determines its moral and legal status. This way of proceeding, which makes determinations of standing dependent on ontological or psychological properties, like consciousness or sentience, has traction in both moral philosophy and law. But it is not the only, or even the best, method for deciding these matters. One recent and promising alternative is relational ethics. The final set of essays investigate the opportunities and challenges of this moral and legal innovation.

In “Empathizing and Sympathizing With Robots: Implications for Moral Standing” Quick employs a phenomenological approach to investigating human-robot interaction (HRI), arguing that empathetic and sympathetic engagements with social robots takes place in terms of and is experienced as an ethical encounter. Following from this, Quick concludes, such artifacts will need to be recognized as another form of socially significant otherness and would therefore be due a minimal level of moral consideration.

With “Robot Responsibility and Moral Community,” Dane Leigh Gogoshin recognizes that the usual way of deciding questions of moral responsibility would certainly exclude robots due to the fact that these technological artifacts lack the standard qualifying properties to be considered legitimate moral subjects, i.e. consciousness, intentionality, empathy, etc. But, Gogoshin argues, this conclusion is complicated by actual moral responsibility practices, where human beings often respond to rule-abiding robots as morally responsible subjects and thus members of the moral community. To address this, Gogoshin proposes alternative accountability structures that can accommodate these other forms of moral agency.

The essay “Does the Correspondence Bias Apply to Social Robots?: Dispositional and Situational Attributions of Human Versus Robot Behavior” adds empirical evidence to this insight. In this essay, human-machine communication researchers Edwards and Edwards investigate whether correspondence bias (e.g. the tendency for individuals to over-emphasize personality-based explanations for other people’s behavior while under-emphasizing situational explanations) applies to social robots. Results from their experimental study indicate that participants do in fact make correspondent inferences when evaluating robots and attribute behaviors of the robot to perceived underlying attitudes even when such behaviors are coerced.

With the essay “On the Social-Relational Moral Standing of AI: An Empirical Study Using AI-Generated Art,” Lima et al. turn attention from the social circumstances of HRI to a specific domain where robot intervention is currently disrupting expected norms. In their social scientific investigation, the authors test whether and how interacting with AI-generated art affects the perceived moral standing of its creator, and their findings provide useful and empirically grounded insights concerning the operative limits of moral status attribution.

Finally, if these three essays provide support for a socially situated form of relational ethics, then the essay from Sætra—“Challenging the Neo-Anthropocentric Relational Approach to Robot Rights”—provides an important counterpoint. Unlike traditional forms of moral thinking where what something is determines how it is treated, relationalism promotes an alternative procedure that flips the script on this entire transaction. In his engagement with the existing literature on the subject, Sætra finds that the various articulations of “relationalism,” despite many advantages and opportunities, might not be able to successfully resolve or escape from the problems that have been identified.

In presenting this diverse set of essays, our intention has been to facilitate and stage a debate about the moral and legal status of social robots that can help theorists and practitioners not only make sense of the current state of research in this domain but also assist them in the development of their own thinking about and research into these important and timely concerns. Consequently, our objective with the Research Topic is not to advance one, definitive solution or promote one way to resolve these dilemmas but to map the range of possible approaches to answering these questions and provide the opportunity for readers to critically evaluate their significance and importance.
